# Association between Cognitive Impairment and Freezing of Gait in Patients with Parkinson’s Disease

**DOI:** 10.3390/jcm12082799

**Published:** 2023-04-10

**Authors:** Yifei Gan, Hutao Xie, Guofan Qin, Delong Wu, Ming Shan, Tianqi Hu, Zixiao Yin, Qi An, Ruoyu Ma, Shu Wang, Quan Zhang, Guanyu Zhu, Jianguo Zhang

**Affiliations:** 1 Department of Neurosurgery, Beijing Tiantan Hospital, Capital Medical University, Beijing 100070, China; 2 Department of Neurosurgery, The First Affiliated Hospital of Anhui Medical University, Jixi Road 218, Hefei 230022, China; 3 Beijing Key Laboratory of Neurostimulation, Beijing 100070, China

**Keywords:** Parkinson’s disease, freezing of gait, cognitive impairment, cognitive heterogeneity, executive function

## Abstract

**Background:** Freezing of gait (FOG) is a common disabling symptom in Parkinson’s disease (PD). Cognitive impairment may contribute to FOG. Nevertheless, their correlations remain controversial. We aimed to investigate cognitive differences between PD patients with and without FOG (nFOG), explore correlations between FOG severity and cognitive performance and assess cognitive heterogeneity within the FOG patients. **Methods:** Seventy-four PD patients (41 FOG, 33 nFOG) and 32 healthy controls (HCs) were included. Comprehensive neuropsychological assessments testing cognitive domains of global cognition, executive function/attention, working memory, and visuospatial function were performed. Cognitive performance was compared between groups using independent *t*-test and ANCOVA adjusting for age, sex, education, disease duration and motor symptoms. The k-means cluster analysis was used to explore cognitive heterogeneity within the FOG group. Correlation between FOG severity and cognition were analyzed using partial correlations. **Results:** FOG patients showed significantly poorer performance in global cognition (MoCA, *p* < 0.001), frontal lobe function (FAB, *p* = 0.015), attention and working memory (SDMT, *p* < 0.001) and executive function (SIE, *p* = 0.038) than nFOG patients. The FOG group was divided into two clusters using the cluster analysis, of which cluster 1 exhibited worse cognition, and with older age, lower improvement rate, higher FOGQ3 score, and higher proportion of levodopa-unresponsive FOG than cluster 2. Further, in the FOG group, cognition was significantly correlated with FOG severity in MoCA (r = −0.382, *p* = 0.021), Stroop-C (r = 0.362, *p* = 0.030) and SIE (r = 0.369, *p* = 0.027). **Conclusions:** This study demonstrated that the cognitive impairments of FOG were mainly reflected by global cognition, frontal lobe function, executive function, attention and working memory. There may be heterogeneity in the cognitive impairment of FOG patients. Additionally, executive function was significantly correlated with FOG severity.

## 1. Introduction

Freezing of gait (FOG), defined as the “brief, episodic absence or marked reduction of forward progression of the feet despite the intention to walk” [1], is one of the most problematic disabling phenomena that affects around 25% of patients with early-stage Parkinson’s disease (PD) [2]. The prevalence of FOG increases with the progression of PD and can reach as high as 63% in advanced PD [3]. This paroxysmal gait disturbance increases falls and relative traumas in PD patients, raises disabilities and mortalities, significantly lowers patients’ quality of life, and overloads caretakers [3,4,5]. Nevertheless, it remains one of the most challenging symptoms of PD [6,7].

Despite decades of effort, the exact pathophysiology of FOG remains unclear due to the complexity of its mechanisms. After all, FOG is not merely a motor symptom, but a compound of interactions among motor, cognition, and mood [7,8,9]. Noticeably, cognitive impairment plays a vital role in the development of FOG. Therefore, the relationship between FOG and cognitive impairment has attracted increasing attention [10].

Previous studies found that FOG patients suffered from more severe impairment in executive function, including conflict resolution [11,12] and set-shifting [13,14]. Furthermore, a randomized controlled trial suggested that cognitive training for PD patients with FOG could improve executive function and reduce the severity of FOG [15]. Based on previous studies, researchers proposed the cognitive model of FOG, which emphasized the conflict resolution deficit could enhance the risk of FOG [10]. Moreover, studies found that FOG patients had more severe impairment in attention [16], visuospatial function, etc. [17]. However, two recent studies focusing on the executive function and attention of FOG patients concluded differently [18,19], suggesting that there were no significant differences in cognitive performance when comparing the cognitive function of patients with FOG and nFOG.

It seems some previous findings were contradicted. Potential reasons for this controversy were as follows. (i) Many previous studies were based on a relatively small sample size. (ii) Confounders that may affect cognition were controlled differently. Previous studies conducted among PD patients have shown that male patients, older age, shorter years of education, longer disease duration, elevated Hoehn and Yahr stages (H&Y), or higher score on the Movement Disorder Society Unified Parkinson’s Disease rating scale Part III (MDS-UPDRS-III) may lead to cognitive dysfunction [18,19,20,21,22,23]. Thus, we believe that it is necessary to control the above-mentioned confounders at baseline and during the analysis. (iii) FOG is a highly heterogenous motor symptom with variable clinical characteristics [8]. Therefore, we hypothesized that significant cognitive differences might exist among different FOG patients, which means that some FOG patients might have more severe cognitive impairments than others. However, studies focusing on cognitive impairments among FOG individuals are still missing to date.

Therefore, this study aimed to (i) explore the difference in cognitive impairment between FOG patients and nFOG patients while controlling for confounders that may affect cognition, including age, sex, years of education, disease duration, H&Y, and MDS-UPDRS-III; age, sex, years of education and disease duration would be matched at baseline, while H&Y and MDS-UPDRS-III would be controlled using ANCOVA during statistical analysis; (ii) explore whether heterogeneity of cognitive impairments exists within the FOG group; and (iii) explore the correlations between cognition and FOG severity.

## 2. Materials and Methods

### 2.1. Participants

Patients diagnosed with idiopathic PD and age- and sex-matched healthy controls (HC) were recruited from 1 January 2020 to 1 June 2022 at Beijing Tiantan Hospital. Patients with PD were all inpatients from the functional neurosurgery department. Patients were screened by a movement disorders specialist and were included in our study if they (i) met UK Brain Bank diagnostic criteria for idiopathic PD [24]; (ii) were aged between 50 and 80 years; (iii) were systematically educated for more than 5 years; (iv) were on optimized medication regimens for more than 3 months. HCs were included if they were aged between 50 and 80 years. Exclusion criteria for all participants included dementia, severe psychiatric disturbances, vision and hearing abnormalities, history of stroke, brain tumor/traumatic surgery, or supportive brain magnetic resonance imaging (MRI) markers of other neurodegenerative disorders, e.g., multiple system atrophy (MSA) [25,26] and progressive supranuclear palsy (PSP) [27]. All PD participants received at least one brain MRI scan that included 3D-T1 weighted images, and 3D-T2 weighted images for differential diagnosis. All participants provided written informed consent, and the study was approved by the Institutional Review Board of Beijing Tiantan Hospital (IRB number: KY 2018-008-01).

### 2.2. Clinical Assessments

Demographic information including age, sex, and years of education of all participants was collected. For patients with PD, disease duration, levodopa equivalent daily doses (LEDD), Hoehn and Yahr stage (H&Y), and MDS-UPDRS-III scores in ON and OFF state were recorded, and the improvement rate was calculated as [MDS-UPDRS-III (OFF) − MDS-UPDRS-III (ON)]/MDS-UPDRS-III(OFF) ×100%.

### 2.3. Classifications of FOG

Patients with PD were classified into freezing of gait (FOG) if they had a score of more than 1 on the Freezing of Gait Questionnaire (FOGQ) item 3 [28] and scored at least 1 on MDS-UPDRS-III item 11 (MDS-UPDRS-III.11) in the medication withdrawal (OFF) state [26]. The FOG group was further classified as levodopa-responsive FOG (RFOG) and levodopa-unresponsive FOG (URFOG), based on the MDS-UPDRS-III item 11 in medication withdrawal (OFF) and medication (ON) states [29,30]. Briefly, FOG patients who had an improvement of more than one point on item 11 in the ON state compared to the OFF state were classified into RFOG. FOG patients who had an improvement of 1 point or less on item 11 in the ON state were classified into URFOG.

“OFF” assessments were performed with dopaminergic medication withdrawal for at least 12 h, and “ON” assessments were performed 1 h after patients received their first dose of dopaminergic medications on the same day.

### 2.4. Neuropsychological Assessments

All participants received comprehensive neuropsychological assessments including global cognition, frontal lobe function, attention and working memory, executive function, memory, visuospatial function, and language. PD participants were assessed in ON states. Global cognition was evaluated with the Montreal Cognitive Assessment (MoCA, Beijing version). Frontal lobe function was evaluated by the Frontal Assessment Battery (FAB). Attention and working memory were evaluated by the time to complete Trail Marking Test Part A (TMT-A), Stroop Color-Word Test Part B (Stroop-B), the score of Symbol Digit Modalities Test (SDMT), Digit Span Test Forward (DST-F), Digit Span Test Backward (DST-B) and Digit Orientation Test (DOT). Executive function was evaluated with the time to complete Stroop Color-Word Test part C (Stroop-C), Stroop Interference Effect (SIE) calculated by [time to complete Stroop-C − (time to complete Stroop-A + time to complete Stroop-B)/2] [31], and time to complete TMT-B and TMT B-A calculated by time to complete TMT-B minus time to complete TMT-A. Memory was evaluated by the score of the Auditory Verbal Learning Test part 5 (AVLT-5) and AVLT part 7 (AVLT-7). Visuospatial function was assessed by the score of Benton Judgement of Line Orientation (JLO) and Clock Drawing Test (10-point rating scale) (CDT-10). Language was assessed by the score of the Boston Naming Test (BNT) and the Verbal Fluency Test, consisting of an animal fluency test (aVFT), a household items fluency test (hVFT), and switching between two categories (sVFT). Anxiety and depression were evaluated by the Hamilton Anxiety Rating Scale (HAMA, 14-item version) [32] and the Hamilton Depression Rating Scale (HAMD, 24-item version) [33].

### 2.5. Statistical Analysis

Demographic and clinical characteristics as well as cognitive performance were compared between HC and PD, and between FOG and nFOG using independent samples *t*-test and chi-square tests where appropriate. Analysis of covariance (ANCOVA) adjusting for covariates including age, sex, years of education, years of disease duration, and the score of MDS-UPDRS-III was also conducted when comparing cognitive performance between HC and PD, and between FOG and nFOG.

To explore the cognitive heterogeneity within the FOG group, K-means cluster analyses were conducted. MoCA, FAB, SDMT, and SIE were used as clustering variables due to their significant differences after adjusting for covariates in comparisons between FOG and nFOG. Eventually, the four distinguishable variables divided the FOG group into two clusters, in which cluster 1 had worse cognitive performance compared with cluster 2. For further analysis, we compared the demographic, clinical, and cognitive characteristics between the two clusters using the independent *t*-test, Mann–Whitney U test, and chi-square test when appropriate.

To determine the association between the severity of FOG and cognition, partial correlation controlling for age, sex, years of education, years of disease duration, and MDS-UPDRS-III (ON) were performed between FOGQ and cognitive performance within the FOG group and the two FOG clusters. All analyses were performed using SPSS 28.0 (IBM, Chicago, IL, USA).

## 3. Results

### 3.1. Demographic and Clinical Features

The demographics and clinical features are detailed in Table 1. A total of 106 participants (74 PD, 32 HCs) were included in this study. Of those with PD, 41 were classified as FOG. PD patients and HCs, as well as FOG and nFOG, were matched for age, sex, and years of education. FOG and nFOG patients did not differ in terms of LEDD, onset sides, improvement rate, HAMA, and HAMD. However, the H&Y stage (*p* = 0.033) and MDS-UPDRS-III scores (both ON and OFF state, *p* = 0.001 and *p* < 0.001, respectively) were significantly greater in the FOG group.

### 3.2. Cognitive Performance Comparisons between Groups

The details of differences in cognitive performance between groups are given in Table 2 and Table 3. Before adjusting for covariates, the PD group had worse cognitive performance relative to the HC group for all cognitive tests except for AVLT-5 (*p* = 0.054). After adjusting for age, sex, and years of education, the PD group performed worse than the HC group for all cognitive tests. In addition, the FOG group performed worse in global cognition (MoCA, *p* < 0.001), frontal lobe function (FAB, *p* = 0.010), attention and working memory (SDMT, *p* = 0.001; DOT, *p* = 0.020), executive function (Stroop-C, *p* = 0.026; SIE, *p* = 0.016), visuospatial function (JLO, *p* = 0.029) and language (hVFT, *p* = 0.020; sVFT, *p* = 0.033) relative to the nFOG group. After adjusting for age, sex, years of education, years of disease duration, and MDS-UPDRS-III (ON), fewer differences resulted between the FOG and nFOG groups, with the FOG group showing poorer performance in global cognition (MoCA, *p* < 0.001), frontal lobe function (FAB, *p* = 0.015), attention and working memory (SDMT, *p* < 0.001) and executive function (SIE, *p* = 0.038).

### 3.3. Cluster Analysis

**Figure 1:**
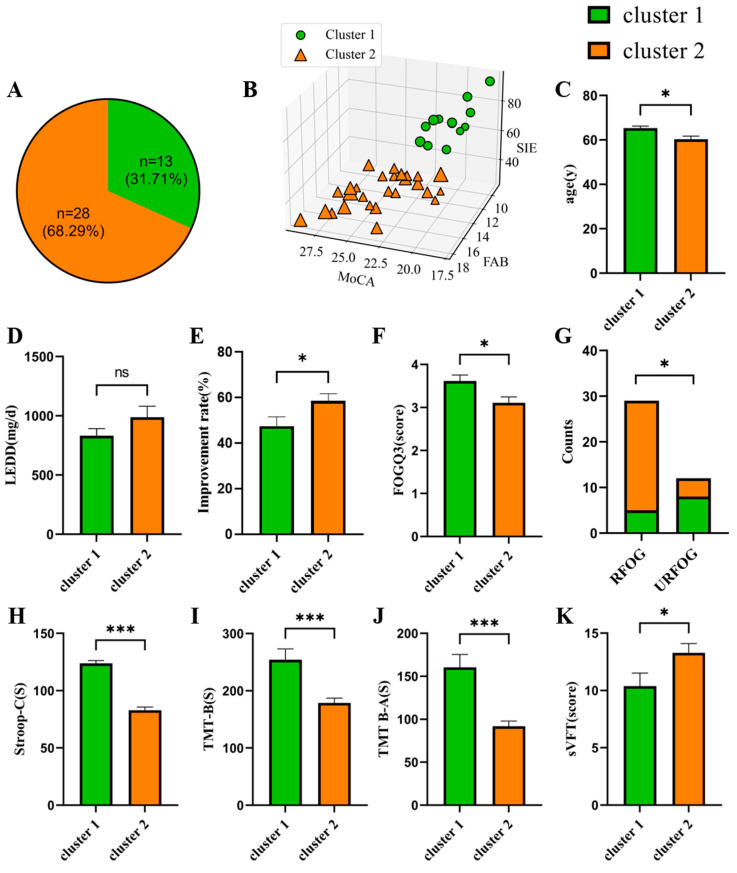
Results of cluster analysis in FOG and comparisons of the two clusters. (**A**) Pie charts showing proportions of clusters in FOG. (**B**) Results of cluster analysis are displayed using differentiation including MoCA, FAB and SIE, and the size of shapes (green circles and orange rectangles) represent the score of SDMT. (**C**) The differences in age between the two clusters (independent *t*-test). (**D**–**G**) The differences in clinical assessments between the two clusters: (**D**,**E**) independent *t*-test; (**F**) Mann–Whitney U test; (**G**) Chi-square test). (**H**–**K**) The differences in cognitive performance between the two clusters (independent *t*-test). *p* values shown represent an analysis of difference between two clusters, * *p* < 0.05; *** *p* < 0.001; ns: *p* > 0.05, not significant (two-tailed). Error bar represents standard error of mean. Abbreviations: FOG = PD with freezing of gait, LEDD = levodopa equivalent daily doses. Improvement rate was calculated as follows: [MDS-UPDRS-III(OFF) − MDS-UPDRS-III (ON)]/MDS-UPDRS-III(OFF)*100%, FOGQ3 = item 3 of FOGQ, RFOG = levodopa-responsive FOG, URFOG = levodopa-unresponsive FOG, Stroop-C = Stroop Part C. SIE = Stroop Interference Effect. TMT-B = TMT Part B. TMT B-A = the time to complete TMT-B minus the time to complete TMT-A. AVLT-5 = Auditory Verbal Learning Test Part 5. sVFT = VFT (switching between category of animals and household items).

We applied the k-means clustering algorithm, to divide FOG according to the cognitive performance of MoCA, FAB, SDMT, and SIE. Then, FOG patients were divided into cluster 1 (*n* = 13) and cluster 2 (*n* = 28, Figure 1A). On average, cluster 1 performed worse in MoCA (F = 28.10, *p* < 0.001), FAB (F = 17.52, *p* < 0.001), SDMT (F = 14.13, *p* < 0.001) and SIE (F = 179.2, *p*< 0.001, Figure 1B). For demographic features, cluster 1 was older (65.31 ± 3.33) relative to cluster 2 (60.32 ± 7.30, *p* = 0.024, Figure 1C). For clinical features, although there were no significant differences in FOGQ between the two clusters, cluster 1 had a lower improvement rate (47.41 ± 14.95% vs. 58.63 ± 16.68%, *p* = 0.045, Figure 1E) and higher FOGQ3 score (3.62 ± 0.51 vs. 3.11 ± 0.74, *p* = 0.041, Figure 1F) than cluster 2. Moreover, we also found a higher proportion of URFOG in cluster 1 (61.54%) versus cluster 2 (14.29%, *p* = 0.002, Figure 1G). For cognitive performance, we also observed that cluster 1 performed worse on Stroop-C (123.80 ± 9.22 vs. 82.93 ± 14.63, *p* < 0.001, Figure 1H), TMT-B (254.10 ± 68.43 vs. 178.80 ± 44.92, *p* < 0.001, Figure 1I), TMT B-A (160.50 ± 53.74 vs. 91.86 ± 32.46, *p* < 0.001, Figure 1J) and sVFT (10.38 ± 4.09 vs. 13.29 ± 4.29, *p* = 0.016, Figure 1K) than cluster 2.

### 3.4. Association between FOG Severity and Cognition

We presented the results of the correlation analysis in Table 4. In the FOG group, cognitive performance was significantly correlated with the severity of FOG measured by FOGQ in MoCA (partial correlation r = −0.382, *p* = 0.021), Stroop-C (partial correlation r = 0.362, *p* = 0.030) and SIE (partial correlation r = 0.369, *p* = 0.027) when controlling for covariates including age, sex, years of education, years of disease duration and MDS_UPDRS_III (ON). In cluster 1, no significant correlations were found between cognitive performance and FOGQ. In cluster 2, cognitive performance including global cognition (MoCA, r = −0.509, *p* = 0.013) and executive function (Stroop-C, r = 0.488, *p* = 0.188; SIE, r = 0.636, *p* = 0.001) were significantly correlated with FOGQ after controlling for age, sex, years of education, years of disease duration and MDS-UPDRS-III (ON).

## 4. Discussion

In this study, we performed comprehensive neuropsychological assessments in PD patients with and without FOG, as well as healthy controls. The results from comparative analyses indicated that the FOG group performed worse in global cognition, frontal lobe function, executive function, attention and working memory than the nFOG group. Through cluster analysis, the FOG group was divided into two clusters according to the scores of MoCA, FAB, SDMT, and SIE. Patients in cluster 1 performed worse in executive function compared to those in cluster 2. This suggested that heterogeneity of cognitive impairments may exist within the FOG group. In addition, the correlations between FOG severity and cognitive performance including global cognition and executive function could be observed in both the FOG group and cluster 2.

### 4.1. Cognitive Dysfunction and FOG

In line with most of the previous studies [7,10,11,12,13,14,34,35], the present study showed that compared to nFOG patients, FOG patients performed worse in global cognition, frontal lobe function, executive function, attention and working memory when controlling for factors affecting cognitive function. Shine et al. [36] utilized a virtual reality (VR) task stimulating walking scenarios for PD patients with FOG, while functional magnetic resonance imaging (fMRI) data were collected for a series of explorations on the pathophysiology of FOG. The team revealed that increased response in frontoparietal regions was negatively correlated with the severity of FOG symptoms [37], and during freezing episodes, significantly decreased activations of basal ganglia, thalamus, and the pre-supplementary motor area (pre-SMA) were observed [38]. The above-mentioned brain regions also participate in executive function [39]. Further analyses based on the functional connectivity of VR tasks revealed that PD patients with FOG (in the medication-withdrawal state) showed functional coupling between the cognitive control network (including the dorsolateral prefrontal cortex and the posterior parietal cortex) and the basal ganglia networks [40]. These findings suggest that the occurrence of freezing may relate to the decreased functional connectivity between the frontoparietal network (responsible for goal-oriented behavior, which is an executive function) and basal ganglia network (responsible for automatic gait control). In line with the studies by Shine et al. [36,37,38,40], similar conclusions were drawn by two other studies [41,42] that revealed the presence of the decoupling between the cognitive control network and striatum in PD patients with FOG. Thus, we inferred that cognitive dysfunction, especially executive dysfunction caused by the decoupling between the cognitive control network and striatum, may promote the occurrence of FOG.

Recently, a novel study based on a large sample was reported by Morris et al. [18]. They compared global cognition, executive function/attention, working memory, and visuospatial function between a FOG group (*n* = 66) and an nFOG group (*n* = 81). No significant difference was found when controlling for covariates. Here, we concluded differently from their findings, and the possible explanations are as follows: (1) Morris et al. [18] performed cognitive assessments under OFF state, while our assessments were performed under ON state, controlling for motor symptoms; (2) FOG patients in our study had longer years of disease duration compared to theirs (9.21 ± 3.30 vs. 7.83 ± 3.81); (3) heterogeneity of cognitive impairment may exist in the FOG group, which can be stratified by different freezing triggers such as motor type (freezing when turning), cognitive type (freezing when dual tasking) or limbic type (freezing when anxious) [8]. Additionally, executive function differentiated between FOG sub-types based on responsiveness to dopamine [29], in which URFOG patients had more severe impairment of executive function compared to RFOG patients. In our study, 13 out of 41 FOG patients were classified as URFOG (29.27%), while the study of Morris et al. [18] did not mention responsiveness to dopamine.

### 4.2. Cognitive Model of FOG

Conflict resolution, which is an important aspect of executive function, was traditionally tested by Stroop, Flanker, and go/no-go tasks [11]. In our study, although the difference in Stroop-C was not significant after controlling for covariates, SIE remained significantly worse in the FOG group than in the nFOG group. Further correlation analysis also showed that the severity of FOG symptoms was correlated with SIE and Stroop-C (reflecting conflict resolution). These results are consistent with the study of Vandenbossche et al. [12], which discovered conflict resolution deficits using the Stroop task and attention network test. In addition, our findings possibly support that the cognitive model of FOG, which emphasizes the conflict resolution deficit, could enhance the risk of FOG [43]. As we mentioned above, previous studies utilizing event-related fMRI have found that the abnormal activation or abnormal functional connectivity of PD patients with FOG is mainly converged on “the hyperdirect pathway” (including the pre-SMA and the subthalamic nucleus) [44]. This allows direct projections from the premotor region to the subthalamic nucleus (STN) without the involvement of the striatum. This “hyperdirect pathway” also participates in conflict resolution and reactive inhibition [39]. Therefore, the weakened functional connectivity between pre-SMA and STN in FOG patients, together with the decreased activation of the pre-SMA in event-related fMRI, might lead to conflicts among series of motor planning due to the interactions of competitions and compensations [45]. This could lead to excessive excitatory output from the STN to the globus pallidus internus (GPi), which further increases the inhibition of GABA-energic neurons of the motor regions, eventually resulting in the occurrence of FOG [46].

### 4.3. Cognitive Heterogeneity in FOG

FOG patients were divided into two clusters using the k-means cluster analysis. MoCA (global cognition), FAB (frontal lobe function), SIE (executive function), and SDMT (attention and working memory) were enrolled as clustering variables. Compared to cluster 2, cluster 1 performed significantly worse in cognitive scales including TMT-B, TMT B-A, Stroop-C, and sVFT other than the four scales used as clustering variables. This indicated that cognitive heterogeneity may exist within the FOG group [8,29]. Moreover, we found that the cognitive differences between clusters were mainly focused on the “frontal” domains based on stronger measures of executive function/attention [47,48], since FAB, SIE, SDMT, TMT-B, TMT B-A, and Stroop-C belonged to tasks of executive function/attention [49]. Although VFT was classified as a test of language in our study, some studies used it as a test of executive function. Previous studies also suggested that VFT was correlated with frontal lobe function [47,50]. Therefore, we speculate that impairment in “frontal” domains may play an important role in FOG, but this may be heterogeneous in FOG individuals. Furthermore, compared to cluster 2, which performed better on cognitive tests, patients from cluster 1 were older, with lower improvement rates and a lower score on FOGQ3. Of note was a higher proportion of URFOG in cluster 1 (61.54%) relative to cluster 2. As we mentioned above, URFOG patients had more severe executive function impairment [29].

### 4.4. Limitations

There are some limits to our study. First, this was an observational, single-center study, and the sample size needs to be further expanded. Second, misclassification of PD diagnosis may be present, as confirmation of diagnosis by means of a dopamine transporter (DAT)-PET/SPECT was not available. Third, FOG was defined by FOGQ, which is a self-reported questionnaire and mainly depends on patients’ subjective recall. Although FOGQ has been widely used to assess the severity of FOG and has been considered a reliable screening tool for FOG patients [28], in future studies, we would apply a more objective and quantifiable method for FOG symptom monitoring, such as 2-min 360 degrees turning in place [51]. In addition, cognitive assessments of our study were conducted under the medication “ON” state in order to minimize the effect of motor symptoms on cognition. However, dopaminergic medication has a complex effect on cognitive function, and we cannot exclude its effects on cognition [52,53,54].

## 5. Conclusions

This study highlights that FOG patients have poorer cognitive performance on global cognition, frontal lobe function, attention and working memory, and executive function than nFOG patients. Furthermore, the cluster analysis suggests that cognitive heterogeneity may exist within the FOG group, and there may be differences among FOG individuals. We also found significant correlations between FOG severity and executive function. All in all, our findings add new evidence to cognitive models of FOG, which may shed new light on FOG treatment in the future.

## Figures and Tables

**Table 1:** Demographic and clinical characteristics of all participants.

	HC (*n* = 32)	PD (*n* = 74)	FOG (*n* = 41)	nFOG (*n* = 33)	*p*-Value of Independent *t*-Test	
					HC v PD	FOG v nFOG
Age (y)	60.84 ± 6.04	61.81 ± 7.01	61.90 ± 6.69	61.70 ± 7.48	0.499	0.901
Sex (M/F) ^a^	15/17	43/31	26/15	17/16	0.286	0.302
Education (y)	10.78 ± 2.84	11.06 ± 3.40	10.99 ± 3.84	11.15 ± 2.82	0.685	0.833
Disease duration (y)	NA	8.63 ± 3.25	9.21 ± 3.30	7.91 ± 3.09	NA	0.088
LEDD (mg/d)	NA	869.15 ± 406.62	938.57 ± 423.70	782.9 ± 372.75	NA	0.102
H&Y (2/2.5/3/4) ^a^	NA	4/11/48/11	1/4/27/10	3/7/21/1	NA	0.033 *
Onset side (B/R/L)	NA	5/44/25	3/25/14	2/19/11	NA	0.984
MDS-UPDRS-III (ON)	NA	19.81 ± 11.02	23.10 ± 12.26	15.73 ± 7.63	NA	0.001 *
MDS-UPDRS-III (OFF)	NA	44.74 ± 16.84	50.95 ± 17.12	37.03 ± 13.04	NA	<0.001 *
MDS-UPDRS-III.11 (ON)	NA	0.49 ± 0.90	0.88 ± 1.05	0 ± 0	NA	<0.001 *
MDS-UPDRS-III.11 (OFF)	NA	1.46 ± 1.45	2.63 ± 0.83	0 ± 0	NA	<0.001 *
Improvement rate (%)	NA	56 (15.9)	55 (16.8)	58 (14.8)	NA	0.428
RFOG/URFOG	NA	28/13	28/13	NA	NA	NA
FOGQ	NA	11.12 ± 8.49	17.95 ± 3.89	2.64 ± 3.41	NA	<0.001 *
FOGQ3 ^b^	NA	1.91 ± 1.65	3.27 ± 0.71	0.21 ± 0.49	NA	<0.001 *
HAMA	NA	15.80 ± 9.08	15.78 ± 8.75	15.82 ± 9.61	NA	0.986
HAMD	NA	15.97 ± 8.51	16.46 ± 8.76	15.36 ± 8.29	NA	0.582

Values are presented with means ± standard deviations or frequencies. ^a^ Chi-square test, ^b^ Nonparametric test using the Mann–Whitney U test, * significant difference. Abbreviations: NA = not applicable, HC = healthy controls, PD = Parkinson’s disease, FOG = PD with freezing of gait, nFOG = PD without freezing of gait, LEDD = levodopa equivalent daily doses, RFOG = levodopa-responsive FOG, URFOG = levodopa-unresponsive FOG, H&Y = Hoehn and Yahr stage, MDS-UPDRS-III (ON) = the Movement Disorder Society Unified Parkinson’s Disease rating scale Part III (on-medication), MDS-UPDRS-III(OFF) = MDS-UPDRS-III (medication-withdrawal), MDS-UPDRS-III.11 (ON) = MDS-UPDRS-III item 11 (on medication), MDS-UPDRS-III.11 (OFF) = MDS-UPDRS-III item 11 (medication-withdrawal). Improvement rate was calculated as follows: [MDS-UPDRS-III(OFF) − MDS-UPDRS-III (ON)]/MDS-UPDRS-III(OFF)*100%, FOGQ = the Freezing of Gait Questionnaire, FOGQ3 = item 3 of FOGQ, HAMA = Hamilton Anxiety Rating Scale(14-item version), HAMD = the Hamilton Depression Rating Scale (24-item version).

**Table 2:** Differences in cognitive performance between HC and PD.

	HC (*n* = 32)	PD (*n* = 74)	*p*-Value of Independent *t*-Test	*p*-Value of ANCOVA ^a^
Global cognition				
MoCA	22.10 ± 3.06	24.91 ± 2.40	<0.001 *	<0.001 *
Frontal lobe function				
FAB	15.24 ± 1.83	16.39 ± 1.87	0.037 *	0.030 *
Attention and working memory				
TMT-A (s)	89.22 ± 30.78	82.24 ± 27.10	<0.001 *	<0.001 *
Stroop-B (s)	44.59 ± 10.56	44.09 ± 12.84	<0.001 *	<0.001 *
SDMT	22.17 ± 9.28	29.88 ± 8.94	<0.001 *	<0.001 *
DST-F	5.17 ± 1.28	5.72 ± 1.21	0.002 *	0.013 *
DST-B	2.98 ± 1.05	3.41 ± 1.16	<0.001 *	<0.001 *
DOT	4.85 ± 1.68	5.79 ± 1.69	<0.001 *	<0.001 *
Executive function				
Stroop-C (s)	95.87 ± 23.24	82.45 ± 27.50	<0.001 *	<0.001 *
SIE (s)	57.71 ± 20.95	45.62 ± 20.83	<0.001 *	<0.001 *
TMT-B (s)	202.6 ± 63.45	181.9 ± 68.98	<0.001 *	<0.001 *
TMT B-A (s)	113.6 ± 51.21	99.64 ± 53.12	0.026 *	0.024 *
Memory				
AVLT-5	5.07 ± 2.09	5.18 ± 3.15	0.054	0.019 *
AVLT-7	21.51 ± 1.90	21.82 ± 1.80	0.020 *	0.023 *
Visuospatial function				
JLO	19.10 ± 4.38	20.06 ± 3.89	0.029 *	0.012 *
CDT-10	8.13 ± 1.67	8.91 ± 2.01	<0.001 *	<0.001 *
Language				
Boston naming test	25.80 ± 2.27	26.73 ± 1.92	0.038 *	0.015 *
aVFT	15.90 ± 5.32	17.45 ± 4.99	<0.001 *	<0.001 *
hVFT	14.49 ± 4.51	17.21 ± 5.36	<0.001 *	<0.001 *
sVFT	12.37 ± 4.40	14.67 ± 4.67	0.001 *	0.001 *

Values are presented with means ± standard deviations. ^a^ Controlling for age, sex, years of education. * significant difference. Abbreviations: HC = healthy controls, PD = Parkinson’s disease. ANCOVA = analysis of covariance. MoCA = Montreal Cognitive Assessment (Beijing version). FAB = Frontal Assessment Battery. TMT-A = Trail Marking Test part A. Stroop-B = Stroop Color-Word Test Part B. SDMT = Symbol Digit Modalities Test. DST-F = Digit Span Test Forward. DST-B = DST Backward. DOT = Digit Ordering Test. Stroop-C = Stroop Part C. SIE = Stroop Interference Effect. TMT-B = TMT Part B. TMT B-A = the time to complete TMT-B minus the time to complete TMT-A. AVLT-5 = Auditory Verbal Learning Test Part 5. AVLT-7 = AVLT Part 7. JLO = Benton Judgement of Line Orientation. CDT-10 = Clock Drawing Test (10-point rating scale). aVFT = Verbal Fluency Test (category of animals), hVFT = VFT (category of household items), sVFT = VFT (switching between category of animals and household items).

**Table 3:** Differences in cognitive performance between FOG and nFOG.

	FOG (*n* = 41)	nFOG (*n* = 33)	*p*-Value of Independent *t*-Test	*p*-Value of ANCOVA ^a^
Global cognition				
MoCA	22.10 ± 3.06	24.91 ± 2.40	<0.001 *	<0.001 *
Frontal lobe function				
FAB	15.24 ± 1.83	16.39 ± 1.87	0.010 *	0.015 *
Attention and working memory				
TMT-A (s)	89.22 ± 30.78	82.24 ± 27.10	0.310	0.491
Stroop-B (s)	44.59 ± 10.56	44.09 ± 12.84	0.856	0.199
SDMT	22.17 ± 9.28	29.88 ± 8.94	0.001 *	<0.001 *
DST-F	5.17 ± 1.28	5.72 ± 1.21	0.061	0.365
DST-B	2.98 ± 1.05	3.41 ± 1.16	0.096	0.319
DOT	4.85 ± 1.68	5.79 ± 1.69	0.020 *	0.133
Executive function				
Stroop-C (s)	95.87 ± 23.24	82.45 ± 27.50	0.026 *	0.123
SIE (s)	57.71 ± 20.95	45.62 ± 20.83	0.016 *	0.038 *
TMT-B (s)	202.6 ± 63.45	181.9 ± 68.98	0.183	0.168
TMT B-A (s)	113.6 ± 51.21	99.64 ± 53.12	0.255	0.172
Memory				
AVLT-5	5.07 ± 2.09	5.18 ± 3.15	0.859	0.229
AVLT-7	21.51 ± 1.90	21.82 ± 1.80	0.786	0.681
Visuospatial function				
JLO	19.10 ± 4.38	20.06 ± 3.89	0.327	0.078
CDT-10	8.13 ± 1.67	8.91 ± 2.01	0.028 *	0.093
Language				
Boston naming test	25.80 ± 2.27	26.73 ± 1.92	0.067	0.091
aVFT	15.90 ± 5.32	17.45 ± 4.99	0.204	0.555
hVFT	14.49 ± 4.51	17.21 ± 5.36	0.020 *	0.057
sVFT	12.37 ± 4.40	14.67 ± 4.67	0.033 *	0.217

Values are presented with means ± standard deviations. ^a^ Controlling for age, sex, years of education, disease duration, MDS-UPDRS-III (ON). * Significant difference. Abbreviations: FOG = PD with freezing of gait. nFOG = PD without freezing of gait. ANCOVA = analysis of covariance. MoCA = Montreal Cognitive Assessment (Beijing version). FAB = Frontal Assessment Battery. TMT-A = Trail Marking Test part A. Stroop-B = Stroop Color-Word Test Part B. SDMT = Symbol Digit Modalities Test. DST-F = Digit Span Test Forward. DST-B = DST Backward. DOT = Digit Ordering Test. Stroop-C = Stroop Part C. SIE = Stroop Interference Effect. TMT-B = TMT Part B. TMT B-A = the time to complete TMT-B minus the time to complete TMT-A. AVLT-5 = Auditory Verbal Learning Test Part 5. AVLT-7 = AVLT Part 7. JLO = Benton Judgement of Line Orientation. CDT-10 = Clock Drawing Test (10-point rating scale). aVFT = Verbal Fluency Test (category of animals), hVFT = VFT (category of household items), sVFT = VFT (switching between category of animals and household items).

**Table 4:** Correlation analysis between FOG severity and cognitive performance in patients with FOG.

Cognitive Performance	FOGQ in Cluster 1 (*n* = 13)		FOGQ in Cluster 2 (*n* = 28)		FOGQ in FOG (*n* = 41)	
	Uncontrolled	Controlled ^a^	Uncontrolled	Controlled ^a^	Uncontrolled	Controlled ^a^
Global cognition						
MoCA	−0.476 (0.100)	−0.657 (0.077)	−0.397 (0.036 *)	−0.509 (0.013 *)	−0.374 (0.016 *)	−0.382 (0.021 *)
Frontal lobe function						
FAB	−0.116 (0.706)	−0.248 (0.554)	−0.259 (0.183)	−0.291 (0.178)	−0.234 (0.14)	−0.190 (0.267)
Attention and working memory						
TMT-A (s)	0.084 (0.784)	−0.241 (0.565)	0.160 (0.416)	0.227 (0.297)	0.149 (0.352)	0.158 (0.358)
Stroop-B (s)	−0.174 (0.569)	−0.484 (0.224)	0.026 (0.897)	0.022 (0.919)	−0.004 (0.982)	−0.019 (0.912)
SDMT	0.001 (0.996)	0.357 (0.386)	−0.024 (0.904)	−0.200 (0.359)	−0.078 (0.628)	−0.088 (0.608)
DST-F	−0.093 (0.761)	0.143 (0.735)	−0.087 (0.661)	−0.092 (0.677)	−0.074 (0.648)	−0.037 (0.830)
DST-B	0.325 (0.278)	0.477 (0.232)	−0.038 (0.848)	−0.122 (0.581)	0.003 (0.986)	0.021 (0.903)
DOT	−0.259 (0.392)	−0.129 (0.761)	−0.112 (0.572)	−0.168 (0.442)	−0.169 (0.289)	−0.190 (0.266)
Executive function						
Stroop-C (s)	0.549 (0.052)	0.371 (0.365)	0.442 (0.019 *)	0.488 (0.018 *)	0.353 (0.023 *)	0.362 (0.030 *)
SIE (s)	0.679 (0.011 *)	0.559 (0.150)	0.566 (0.002 *)	0.636 (0.001 *)	0.351 (0.024 *)	0.369 (0.027 *)
TMT-B (s)	−0.151 (0.623)	−0.218 (0.604)	0.197 (0.316)	0.237 (0.276)	0.120 (0.453)	0.121 (0.483)
TMT B-A (s)	−0.224 (0.463)	−0.128 (0.763)	0.122 (0.536)	0.129 (0.557)	0.066 (0.683)	0.065 (0.707)
Memory						
AVLT−5	−0.131 (0.671)	0.360 (0.381)	−0.217 (0.267)	−0.238 (0.273)	−0.209 (0.190)	−0.129 (0.453)
AVLT−7	0.392 (0.186)	0.469 (0.241)	−0.133 (0.500)	−0.139(0.528)	0.002 (0.992)	0.030 (0.863)
Visuospatial function						
JLO	−0.016 (0.959)	−0.229 (0.586)	−0.020 (0.918)	−0.022 (0.921)	−0.035 (0.828)	0.004 (0.982)
CDT−10	0.161 (0.598)	0.204 (0.628)	−0.001 (0.998)	−0.146 (0.506)	0.049 (0.760)	0.063 (0.717)
Language						
Boston naming test	−0.057 (0.854)	0.012 (0.977)	−0.183 (0.351)	−0.283 (0.192)	−0.148 (0.355)	−0.202 (0.238)
aVFT	−0.013 (0.965)	−0.310 (0.455)	−0.260 (0.181)	−0.396 (0.061)	−0.197 (0.216)	−0.215 (0.208)
hVFT	−0.027 (0.931)	−0.164 (0.697)	−0.145 (0.463)	−0.215 (0.325)	−0.139 (0.388)	−0.120 (0.486)
sVFT	0.296 (0.326)	0.339 (0.411)	−0.049 (0.804)	−0.049 (0.823)	−0.004 (0.980)	0.053 (0.757)

Presented values are Pearson’s correlation coefficients and *p*-values (in brackets). ^a^ Controlling for age, sex, years of education, disease duration and MDS-UPDRS-III (ON). * Significant difference. Abbreviations: FOG = Parkinson’s disease with freezing of gait. FOG1 = FOG type 1. FOG2 = FOG type 2. MoCA = Montreal Cognitive Assessment (Beijing version). FAB = Frontal Assessment Battery. TMT-A = Trail Marking Test Part A. Stroop-B = Stroop Color-Word Test Part B. SDMT = Symbol Digit Modalities Test. DST-F = Digit Span Test Forward. DST-B = DST Backward. DOT = Digit Ordering Test. Stroop-C = Stroop Part C. SIE = Stroop Interference Effect. TMT-B = TMT Part B. TMT B-A = the time to complete TMT-B minus the time to complete TMT-A. AVLT-5 = Auditory Verbal Learning Test Part 5. AVLT-7 = AVLT Part 7. JLO = Benton Judgement of Line Orientation. CDT-10 = Clock Drawing Test (10-point rating scale). aVFT = Verbal Fluency Test (category of animals), hVFT = VFT (category of household items), sVFT = VFT (switching between category of animals and household items).

## Data Availability

The data presented in this study are available on request from the corresponding author. The data are not publicly available due to privacy regulations regarding patients.
